# A fast extraction-free isothermal LAMP assay for detection of SARS-CoV-2 with potential use in resource-limited settings

**DOI:** 10.1186/s12985-022-01800-7

**Published:** 2022-05-02

**Authors:** Kathleen Gärtner, Harry Meleke, Mercy Kamdolozi, David Chaima, Lyson Samikwa, Mary Paynter, Maggie Nyirenda Nyang’Wa, Elaine Cloutman-Green, Eleni Nastouli, Nigel Klein, Tonney Nyirenda, Chisomo Msefula, Dagmar G. Alber

**Affiliations:** 1 Great Ormond Street Institute of Child Health, University College London, London, UK; 2 Pathology Department, Kamuzu University of Health Sciences, Blantyre, Malawi; 3 St. George’s University Hospitals London, London, UK; 4 Great Ormond Street Hospital, NHS Foundation Trust, London, UK; 5 University College London Hospital, London, UK

**Keywords:** SARS-CoV-2, qRT-PCR, Extraction-free LAMP, Resource-limited settings

## Abstract

**Background:**

To retain the spread of SARS-CoV-2, fast, sensitive and cost-effective testing is essential, particularly in resource limited settings (RLS). Current standard nucleic acid-based RT-PCR assays, although highly sensitive and specific, require transportation of samples to specialised laboratories, trained staff and expensive reagents. The latter are often not readily available in low- and middle-income countries and this may significantly impact on the successful disease management in these settings. Various studies have suggested a SARS-CoV-2 loop mediated isothermal amplification (LAMP) assay as an alternative method to RT-PCR.

**Methods:**

Four previously published primer pairs were used for detection of SARS-CoV-2 in the LAMP assay. To determine optimal conditions, different temperatures, sample input and incubation times were tested. Ninety-three extracted RNA samples from St. George's Hospital, London, 10 non-extracted nasopharyngeal swab samples from Great Ormond Street Hospital for Children, London, and 92 non-extracted samples from Queen Elisabeth Central Hospital (QECH), Malawi, which have previously been tested for SARS-Cov-2 by quantitative reverse-transcription RealTime PCR (qRT-PCR), were analysed in the LAMP assay.

**Results:**

In this study we report the optimisation of an extraction-free colourimetric SARS-CoV-2 LAMP assay and demonstrated that a lower limit of detection (LOD) between 10 and 100 copies/µL of SARS-CoV-2 could be readily detected by a colour change of the reaction within as little as 30 min. We further show that this assay could be quickly established in Malawi, as no expensive equipment is necessary. We tested 92 clinical samples from QECH and showed the sensitivity and specificity of the assay to be 86.7% and 98.4%, respectively. Some viral transport media, used routinely to stabilise RNA in clinical samples during transportation, caused a non-specific colour-change in the LAMP reaction and therefore we suggest collecting samples in phosphate buffered saline (which did not affect the colour) as the assay allows immediate sample analysis on-site.

**Conclusion:**

SARS-CoV-2 LAMP is a cheap and reliable assay that can be readily employed in RLS to improve disease monitoring and management.

**Supplementary Information:**

The online version contains supplementary material available at 10.1186/s12985-022-01800-7.

## Background

Two years after the outbreak of the Severe Acute Respiratory Syndrome Coronavirus 2 (SARS-CoV-2) the number of people infected with this new coronavirus is approaching 340 million and more than 5.5 million have died (as of January 2022) [1]. While great international efforts have led to the development and approval of highly effective vaccines against SARS-CoV-2 [2] vaccine-breakthroughs are very common [3, 4], many countries are still seeing high infection rates and are experiencing new waves of infection. To minimise the spread of infection fast isolation of infected individuals as well as efficient and accurate testing is essential. The current gold-standard diagnostic assay for SARS-CoV-2 is a quantitative reverse-transcription PCR (qRT-PCR) assay, which has been developed and optimised in different reference laboratories, including Charité Berlin Germany, CDC China and CDC USA [5–7], and subsequently received approval from the FDA [8].

Fast roll-out of testing was achieved in Europe, North America and Asia with a minimum of > 2000 tests per million individuals to date (December 2021) and Austria even reaching a testing capacity of 46,000 per 10^6^ people. In Africa the testing rate is currently well below 500 tests per million individuals [9], largely because contributing factors include the lack of specialised laboratories and trained staff, insufficient infrastructure for sample transportation and communication of results back to the patient, high costs of the assay and a worldwide shortage of PCR reagents. Therefore, a sensitive, specific and cheap SARS-CoV-2 assay, which does not require the sample to be sent to a specialised laboratory, is urgently needed.

Lateral Flow Tests based on the detection of SARS-CoV-2 antigens have been approved for detection of infections [10]. Although these tests can be used at home they are a lot less sensitive compared to qRT-PCR, with their accuracy being highest when individuals are symptomatic (~ 72% accuracy), decreasing to 58% in asymptomatic people [11]. As this type of test is designed for self-sampling, this adds the risk of inappropriate sample taking by untrained people thus further reducing the accuracy of the test. Therefore, in many countries these tests require additional confirmation by qRT-PCR before a positive test is registered.

The loop-mediated isothermal amplification assay (LAMP) is a rapid and very specific diagnostic assay [12] that can be used as a point-of-care (POC) test and can give results within 20–30 min after taking the sample. Reagents are readily available. The colourimetric assay is based on nucleic acid-amplification and uses the fact that during incorporation of dNTPs into newly synthesised DNA H + ions are released and lead to acidification of the reaction solution. This can be made visible with Phenolred, which turns from a pink colour in basic environments (at the start of the reaction) to a yellow colour in acidic solutions (at the end of the reaction after RNA-amplification) [13]. Fluorescent LAMP assays use a fluorescent dye, which intercalates in double stranded DNA and can be detected, for example, with a light cycler.

For SARS-CoV-2 several LAMP assays have been developed which give reliable results and have received FDA emergency use and authorisation as a POC test [14]. In our study we compared the sensitivity of different SARS-CoV-2 LAMP primers with qRT-PCR and tested the applicability of the assay in Malawi as an example of a resource limited country. We show that the LAMP assay is specific when compared to qRT-PCR. It is a rapid method (30 min), which requires minimal equipment and training, has been successfully tested at Kamuzu College of Health Sciences (KUHeS) in Blantyre, Malawi and its teaching institution Queen Elizabeth Central hospital (QECH). It is cheaper than qRT-PCR and can therefore easily be introduced as POC in resource-limited settings.

## Methods

### Samples and ethical statement

Residual nasopharyngeal and throat swab samples from St. George's Hospital in London, Great Ormond Street Hospital NHS Foundation (GOSH) Trust in London (collected March–May 2020) and QECH in Malawi (collected June–July 2020) were used for LAMP test validation. Ethics approval for KUHeS was obtained from the College of Medicine Research Ethics Committee (COMREC) as part of LAMP assay development studies. Anonymised residual samples originating from the UK were used in accordance with the Human Tissue Act and the RCPath guidelines for assay development and validation.

Non-extracted samples from GOSH were collected in PBS (pH 7.4) and tested within two hours. Non-extracted samples tested in Malawi were collected in viral transport medium. For further analysis samples were frozen at − 80 °C. For the purpose of this study frozen samples were retested by qRT-PCR (N-gene) at the same time as LAMP assays were carried out.

### Aim, design and setting of the study

The aim of the study was to design and optimise a SARS-CoV-2 LAMP assay as rapid test for POC testing in LMICs. At the Institute of Child Health, London, extracted and non-extracted SARS-CoV-2+ samples (identified with qRT-PCR) were used for the optimisation of the LAMP assay. Using the UK qRT-PCR and N-LAMP data in order to achieve 80% power and a significance of *p* < 0.05 a sample size of 72 was required, assuming that 12% of the pairs switched from positive to negative and 0% from negative to positive. We chose 92 non-extracted SARS-CoV-2 samples to be tested retrospectively in Malawi.

### RNA extraction and qRT-PCR

RNA was extracted from 200 µL of swab sample. At KUHeS RNA was extracted using the Omega Biotek Magbind Viral DNA/RNA Kit (Norcross, USA) according to the manufacturer's protocol. The 2019-nCOV CDC EUA Kit primers in combination with qScript™ XLT 1-Step RT-qPCR ToughMix Low ROX mastermix from Quantabio (Beverly, USA) were used for the qRT-PCR. The qRT-PCR was performed on the Quantstudio 7 Flex PCR system.

At St. George's Hospital the Magna Pure 96 DNA and Viral NA Small Volume Kit 2.0 (Roche, Basel, Switzerland) was used with the Pathogen Universal 200 4.0 Extraction protocol (Roche). The extraction volume was 100 µL. For the qRT-PCR 10 µL of extract was tested for SARS-CoV-2 using the Altona Diagnostics Real Star SARS-CoV-2 RT-PCR kit 1.0 on the Roche Light Cycler 480, according to the manufacturer's protocol. Ct values of all samples are shown in Additional file 1: Tables S1 and S2.

### Colourimetric LAMP assay

Primers are listed in Additional file 1: Table S3. The protocol from the New England Biolab (NEB, Ipswich, USA) colourimetric LAMP assay (M1800) was followed with slight modifications to test patient samples. Briefly, all reagents were thawed on ice and pipetted at room temperature. Sample input varied between 1 and 3 µL per 20 µL reactions, which were performed at a temperature range between 59 and 67 °C in a T100 Thermo Cycler (BioRad, Hercules, USA) in London and a GeneAmp PCR System 2700 (Applied Biosystems, Thermo Fisher, Waltham, USA) in Malawi. A change of colour from pink to yellow indicated a positive reaction.

Samples directly tested without prior RNA extraction were heat-inactivated at 95 °C for 5 min before analysis.

Occurrence of LAMP-specific DNA-ladders after the reaction has been tested by agarose gel electrophoresis (1.5%).

### One-step dPCR

Digital droplet PCR (dPCR) was carried out using the Biorad One-Step RT dPCR Supermix and the Biorad Automated Droplet Generator for droplet generation. After the PCR reaction the droplets were read on the QX100 Droplet Reader and results analysed with the QuantaSoft Software (Biorad, Hercules, USA). Primers sequences are listed in Additional file 1: Table S3 and PCR reaction mix and cycling conditions were according to the manufacturer's protocol. Copy numbers of samples were determined by dPCR. To estimate the LOD of the N-LAMP assay samples were serially diluted from 10^4^ to 10^0^ copies per reaction.

### Statistical analysis

For descriptive analysis Cohen’s Kappa was calculated as a measure of agreement between qRT-PCR and LAMP assays. Kappa value ranges from 0 to 1 with 0 indicating no agreement and 1 indicating perfect agreement. The ranges used for interpretation were 0.00–0.20 slight, 0.21–0.40 fair, 0.41–0.60 moderate, 0.61–0.80 substantial and 0.81–1.00 almost perfect agreement.

## Results

### Optimisation of the colourimetric LAMP assay

Four different LAMP primer sets that bind to different regions of the SARS-CoV-2 genome (primer sequences see Additional file 1: Table S3) were tested on extracted SARS-CoV-2 RNA samples that had previously been tested with qRT-PCR at St. George's University Hospital in London (Ct values see Additional file 1: Table S1).

**Fig. 1:**
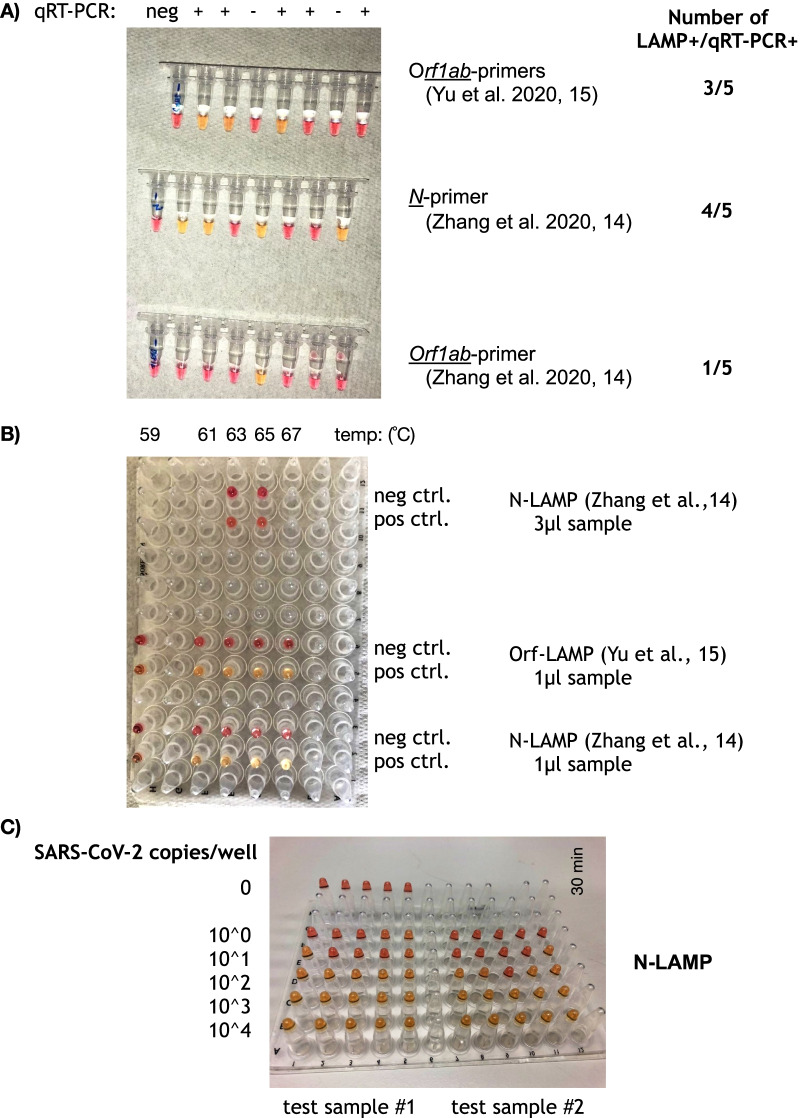
**A** Comparison of different primers on 5 positive and 2 negative qRT-PCR samples (primers taken from different publications as indicated) after 40 min, neg—H_2_O control; **B** Temperature gradient to test LAMP stability, results were recorded after 30 min, primers are from the indicated references; **C** Determination of LAMP sensitivity on a tenfold serial dilution of two samples with known copy numbers (sample #1: 5540 copies/µl, sample #2: 4200 copies/µl)

We chose published primers targeting *orf1a*, *N* [15] and *orf1ab* [16]. Additionally, we also adapted primers from Hong et al., which bind to the *replicase* open reading frame (*orf1ab)* [17] of SARS-CoV-1, to detect SARS-CoV-2. One microliter of five positive and three negative samples (determined by qRT-PCR) were tested at 65 °C following the manufacturer's protocol and colour-change was monitored every ten minutes up to 60 min. The primer set adapted from SARS-CoV-1 [17] did not give any positive results in the colourimetric LAMP assay (data not shown). Figure 1A shows the results for the three published primer sets for SARS-CoV-2 [15, 16]. The best results were obtained with the N-primers, which detected 4 of 5 positive samples, both *orf1* primer sets were less sensitive and detected only 3 of 5 and 1 of 5 positive samples (Fig. 1A). The specificity of the LAMP reaction in SARS-CoV-2 positive samples was shown by the typical ladder-like pattern on an agarose gel (Additional file 2: Fig. S1).

Next, we tested the N-primers in a temperature gradient ranging from 59 to 67 °C, again observing the reaction every 10–60 min to see how the LAMP assay performs at a wider temperature range. At the same time we also compared sample input of 1 and 3 µL at 63 °C and 65 °C. The fastest colour change was seen at 63 °C, appearing after 20 min, closely followed by 65 °C, which is the optimal temperature given by the manufacturer (Fig. 1B). Robust colour change was seen at temperatures between 61 and 67 °C, indicating a relatively wide temperature range at which the LAMP assay can be performed. The lowest temperature of 59 °C did not show a colour change. The amount of sample input also seems to be important, because the colour reaction with 3 µL sample was not as clear as with 1 µL sample, which may indicate inhibitory effects due to large amounts of nucleic acids being present in the reaction.

The ideal condition to perform the LAMP assay seems to be the *N* primer set from Zhang et al. [15] at a temperature of 63 °C for 30–40 min (N-LAMP). Since the *orf1ab* primer set from Yu et al. [16] also performed well (Orf-LAMP) under the same conditions, it was used in parallel also to test clinical samples for the presence of SARS-CoV-2.

To determine the approximate limit of detection of the SARS-CoV-2N-LAMP assay we determined the copy number of two of our samples (sample #1: 5540 copies per µL, sample #2: 4200 copies per µL) in a One-step dPCR reaction using the N2-primers that have been published by the CDC [18]. Both samples were then serially diluted from 10^4^ to 10^0^ copy numbers per reaction and tested in the LAMP assay in five technical replicates. Figure 1C shows that the limit of detection for the N-LAMP assay lies approximately between 10 and 100 copies per reaction.

### Specificity and sensitivity of the colourimetric LAMP assay compared to diagnostic qRT-PCR

**Fig. 2:**
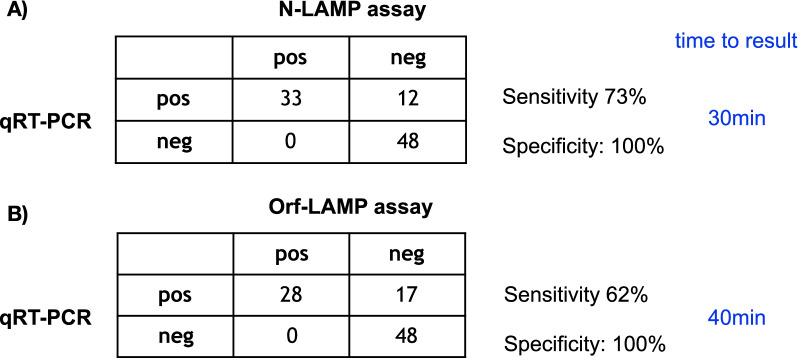
Sensitivity and specificity of LAMP assay compared to qRT-PCR on samples from St. George’s Hospital, London, the crosstables show true positives (pos/pos), true negatives (neg/neg), false positives (neg/pos) and false negatives (pos/neg) of the LAMP assays compared to qRT-PCR; **A** *N* primers and **B** *Orf1ab* primers from Yu et al. [16]

We tested 92 RNA-extracted nasopharyngeal swab samples from St. George's University Hospitals in the LAMP assay that had previously been tested by qRT-PCR using envelope and spike primers as well as qRT-PCR using the CDC N-primers [18]. Figure 2 and Additional file 3: Fig. S2 show that with either the N- or the Orf-LAMP no false-positive samples were detected. Therefore, the specificity of both LAMP assays compared to qRT-PCR was 100% with a confidence interval (CI) of 95.9–100%

The sensitivity of the LAMP assay was found to be slightly lower compared to qRT-PCR (73%, 95% CI: 59.3–84.5% for N-LAMP and 62%, 95% CI: 47.7–75.3% for Orf-LAMP), as both LAMP primer pairs missed some qRT-PCR positive samples (Fig. 2). This was probably due to low amounts of RNA in those samples as shown by a high Ct value of > 30 in the qRT-PCR assays.

The Kappa value was 0.74 (95% CI: 0.61–0.87) and 0.63 (95% CI: 0.48–0.78) for the N- and Orf-LAMP assays, respectively, indicating a significant agreement between each of the LAMP assays and qRT-PCR.

### Use of inactivated non-extracted samples in the colourimetric LAMP assay

**Fig. 3:**
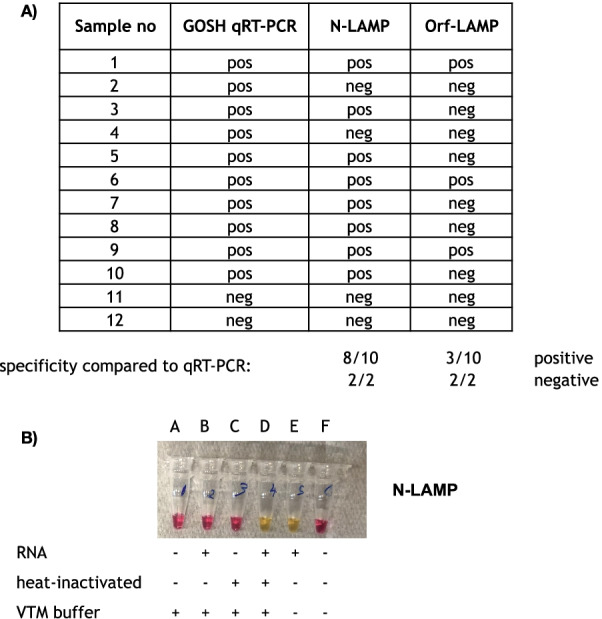
LAMP of non-extracted swab samples from GOSH, London (30 min incubation); **A** N-LAMP and Orf-LAMP results for 12 non-extracted patient samples compared to qRT-PCR, pos—positive results, neg—negative result; **B** N-LAMP assay to test the effect of VTM buffer (conditions A, B, C, D) and heat-inactivation (conditions C, D) on SARS-CoV-2 positive samples (condition B, D, E); + and − indicate presence or absence of the according conditions

To simplify the assay further, shorten the turn-around time and reduce costs we tested the N- and Orf-LAMP assay on non-extracted samples that had previously been tested for SARS-CoV-2 at Great Ormond Street Hospital in London. Ten positive and two negative clinical samples collected in phosphate buffered saline (PBS) were tested using both, the N-LAMP and Orf-LAMP assays (Fig. 3A). The N-LAMP was more sensitive than the Orf-LAMP assay, detecting 8 out of 10 and 3 out of 10 positive samples, respectively. We further tested 35 non-extracted longitudinal swab samples from three patients infected with the SARS-CoV-2 Delta variant. For all positive samples viral nucleic acids could be detected with the N-LAMP assay (data not shown) and the LAMP-typical ladder-like pattern could be confirmed on an agarose gel (Additional file 2: Fig. S1).

### Compatibility of various viral transport media with the LAMP assay

Various viral transport media (VTM) are currently being used worldwide to preserve RNA within clinical specimens during transportation and prior to sample analysis. As the colourimetric LAMP assay measures a pH change caused by the release of H+ ions during the synthesis of new DNA we assessed whether different VTMs on their own would already influence the pH in the reaction mix without incubation at 63 °C. Addition of Universal Transport Medium (UTM, MANTACC), Medical Wire viral medium (MWE) and BDS Sample Preservation Solution were incompatible with the colourimetric LAMP assay, resulting in a colour change from red to yellow immediately after adding to the reaction mix (data not shown). Dewei VTM (Dewei) spiked with SARS-CoV-2 RNA showed inhibition of DNA synthesis (no colour change) in the LAMP reaction, however, heat-inactivation of the Dewei samples for 5 min at 95 °C resulted in a positive reaction seen as colour change from pink to yellow (Fig. 3B). Heat-inactivated samples collected in PBS (pH 7.4) and Beaver VTM (Beaver Biomedical Engineering Co) were also compatible with the LAMP reaction mix (data not shown).

### Testing of the LAMP assay at KUHeS in Malawi

**Fig. 4:**
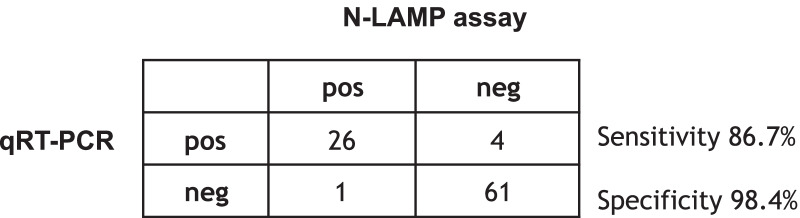
Results for *N*-LAMP testing of non-extracted swab samples at KUHeS, Malawi, the crosstables show true positives (pos/pos), true negatives (neg/neg), false positives (neg/pos) and false negatives (pos/neg) of the LAMP assays compared to qRT-PCR

To assess whether the N-LAMP assay can be used with limited training and equipment in a resource-limited setting (RLS) we tested 92 non-extracted nasopharyngeal and throat swabs with known qRT-PCR result directly on site at KUHeS in Malawi (Ct values see Additional file 1: Table S2). The results are shown in Fig. 4. The LAMP assay performed with a specificity of 98.4% (95% CI: 92.7–99.8%) and a sensitivity of 86.7% (95% CI: 71.3–95.3%), compared to qRT-PCR. The Kappa value is 0.92 (95% CI: 0.77–0.98), which indicates an almost perfect agreement between qRT-PCR and N-LAMP. Again, samples with very high Ct values (above ~ 33) tended to be negative in the N-LAMP assay. Assay inhibition was seen with high amounts of RNA (low Ct-value in qRT-PCR) resulting in a false negative result. Diluting the sample 1:10 resolved this issue and these samples subsequently tested positive by N-LAMP. This confirms that the amount of nucleic acid input into the LAMP assay is important (compare to Fig. 1B).

Interestingly, we found one sample that was negative in qRT-PCR but positive in both, the N-LAMP and Orf-LAMP, in two replicates (Fig. 4 and Additional file 1: Table S2). This was very surprising and this sample needs further characterisation (e.g. sequencing) to determine whether this is a contamination or a mutant that is not detected by qRT-PCR.

## Discussion

The current study confirms that colourimetric SARS-CoV-2 LAMP is a fast, sensitive and reliable assay, which does not require any expensive or bulky equipment. The assay can readily be adapted for use in a resource limited setting (RLS) such as Malawi and therefore could significantly impact on the local SARS-CoV-2 testing capacity.

Although infection rates in Sub-Saharan Africa seem to be lower than elsewhere, testing levels have also generally been lagging behind those in developed economies. This makes clear predictions of the true number of Coronavirus disease 2019 (COVID-19) cases and deaths difficult. A post-mortem study conducted in Zambia showed that due to the lack of SARS-CoV-2 testing, particularly in the wider community, a large number of deaths associated with COVID-19 were missed [19]. Similarly, Mulenga et al. reported that for every 92 SARS-CoV-2 infections in the community only 1 laboratory-confirmed case was reported [20]. Fast, reliable, easy-to-use and affordable SARS-CoV-2 tests are key for monitoring the spread of disease in communities to provide appropriate care, prevent further transmission and allowing the informed management of interventions, such as local lockdowns and implementation of social distancing. SARS-CoV-2 LAMP assays have been described as a low-cost molecular alternative to qRT-PCR [21, 22]. They are now widely used in developed economies as diagnostic tests and as an alternative to qRT-PCR and Lateral Flow Tests, the latter being based on antigen detection and considered less sensitive than nucleic acid-based tests. However, little is known about the utilization of LAMP in RLSs. Baba et al. reported the feasibility of a SARS-CoV-2 LAMP assay in Cameroon, Ethiopia and Nigeria [23]. Their test included a RNA-extraction step, which significantly increases costs, requires a specialised laboratory and lengthens the turn-around-time.

The advantage of the assay described in our study is firstly the direct use of heat-inactivated samples, eliminating lengthy and costly RNA extraction and reducing the risk of infection when samples are handled. Indeed, the assay was more sensitive when samples were not extracted. Although others have suggested that extraction-free LAMP may be less sensitive than LAMP using extracted RNA samples [24], we did not find this in our study. Indeed, similar to our observation, various publications have shown that extraction-free LAMP remains sensitive and specific for its target in the presence of biological fluids, which are known to inhibit qPCR assay [25, 26]. In addition, RNA extraction may result in loss of RNA during the extraction process. Secondly, a water-bath or heat-block is sufficient and as the reaction can tolerate a temperature range from 61 to 67 °C it is less sensitive to temperature fluctuations that may occur in RLSs due to unstable electricity supply. Thirdly, in contrast to standard qRT-PCR, results are available within 30 min after the sample was taken. In addition, reagents are readily available, generally more stable at room temperature for longer time periods than qPCR reagents [26], or indeed could be shipped and stored lyophilised at room temperature [27]. LAMP reagents are much cheaper at a cost of approximately £3 compared to £30 for qRT-PCR.

Of the two primer sets that we tested, the N-LAMP was more sensitive compared to the Orf-LAMP. This is likely due to N RNA being the highest expressed SARS-CoV-2 RNA during virus replication [28].

We found that the amount of input-RNA is critical. High RNA concentration seems to inhibit the reaction, causing false-negative results. To our knowledge, this is the first description of inhibition of a LAMP reaction by very high nucleic acid concentrations. We showed that performing the LAMP assay with undiluted and 1:10 diluted sample resolved this issue.

The SARS-CoV-2 Delta variant could readily be detected by the N-LAMP assay, indicating a higher stability of the assay towards new variants, but further testing and validation of new occurring variants is required. Indeed, similar results have been shown by Promlek et al. [29], who detected SARS-CoV-2 by LAMP during the fourth wave of infection when the dominant strain was Delta.

Limitations of the SARS-CoV-2 LAMP assay include the influence of different viral transport media on the pH of the reaction. Buffers or viral transport media need to be chosen carefully before performing LAMP. We found that collecting samples in PBS is the simplest method, providing the samples are processed fairly quickly. As the LAMP assay is intended to be used as a point-of-care test neither storage nor transportation of the samples is necessary.

The LOD for the LAMP assays to detect SARS-CoV-2 RNA is about 10–100fold higher compared to qRT-PCR ([30] and this work). SARS-CoV-2 RNA can be detected in infected individuals even before symptom onset, which infers that individuals could already spread the virus when still asymptomatic, as well as during the symptomatic phase [31], but the detection of infectious virus seems to wane within the first two to three weeks after infection [31, 32]. During this phase SARS-CoV-2 RNA in patients is very high and LAMP should be more than adequate to detect the virus and especially quarantine asymptomatic people.

## Conclusion

The optimised assay showed similar sensitivity and specificity in Malawi and in London. Future prospective studies in rural health care centres should establish whether this test can be used for wider community surveillance, to inform on adequate disease management.

## Supplementary Information

**Additional file 1**.

**Additional file 2: Figure S1**. Colourimetric N-LAMP assay of positive and negative samples; and gel electrophoresis of colourimetric N-LAMP reaction. Lanes 3, 4, 6 and 7 show the typical ladder pattern of a positive LAMP reaction; 1—H_2_O control, 2—negative sample, extracted RNA, 3,4—positive samples, extracted RNA, 5—negative sample, extracted RNA, 6,7—positive samples, non-extracted RNA, 8—negative sample, non-extracted RNA, L—100 bp DNA ladder

**Additional file 3: Figure S2**. LAMP of 92 samples from St. George’s Hospital in comparison to qRT-PCR results (+/−); **A** N-LAMP (picture taken after 30 min, 63 °C) and **B** Orf-LAMP (picture taken after 40 min, 63 °C); discordant samples are depicted by a red square, − and + indicate negative and positive results in qRT-PCR; pos: positive control, neg: negative control, ** poor qRT-PCR curve/inconclusive

## Data Availability

Original datasets can be obtained from the corresponding author on reasonable request.

## Abbreviations

COVID-19: Coronavirus Disease 19
dPCR: Droplet polymerase chain reaction
LAMP: Loop-mediated isothermal amplification
MWE: Medical wire viral medium
PBS: Phosphate buffered saline
POC: Point-of-care
qRT-PCR: Quantitative reverse transcription polymerase chain reaction
RLS: Resource limited settings
SARS-CoV-2: Severe acute respiratory syndrome coronavirus 2
UTM: Universal transport medium
VTM: Viral transport medium

## References

[1] John’s Hopkins University Coronavirus Resource Centre 2020 [Available from: https://coronavirus.jhu.edu.

[2] Samaranayake LP, Seneviratne CJ, Fakhruddin KS. Coronavirus Disease 2019 (COVID-19) Vaccines: A Concise Review. Oral Dis. 2021. 10.1111/odi.13916 PMC8242875 33991381

[3] Butt AA, Nafady-Hego H, Chemaitelly H, Abou-Samra AB, Khal AA, Coyle PV (2021). Outcomes among patients with breakthrough SARS-CoV-2 infection after vaccination. Int J Infect Dis.

[4] Hacisuleyman E, Hale C, Saito Y, Blachere NE, Bergh M, Conlon EG (2021). Vaccine breakthrough infections with SARS-CoV-2 variants. N Engl J Med.

[5] Institute of Viral Diseases. China CDC. National Institute for Viral Disease Control and Prevention 2020.

[6] Coronavirus Disease 2019 (COVID-19). Centers for Disease Control and Prevention. https://www.cdc.gov/coronavirus/2019-ncov/lab/rt-pcr-panel-primer-probes.html. 2020.

[7] Corman VM, Landt O, Kaiser M, Molenkamp R, Meijer A, Chu DK (2020). Detection of 2019 novel coronavirus (2019-nCoV) by real-time RT-PCR. Euro Surveill.

[8] FDA approved SARS-CoV-2 diagnostic tests 2020 [Available from: https://www.fda.gov/emergency-preparedness-and-response/mcm-legal-regulatory-and-policy-framework/emergency-use-authorization#covidinvitrodev.

[9] https://ourworldindata.org/coronavirus-testing. 2021.

[10] Katyal P, Mahmoudinobar F, Montclare JK (2020). Recent trends in peptide and protein-based hydrogels. Curr Opin Struct Biol.

[11] Dinnes J, Deeks JJ, Berhane S, Taylor M, Adriano A, Davenport C (2021). Rapid, point-of-care antigen and molecular-based tests for diagnosis of SARS-CoV-2 infection. Cochrane Database Syst Rev.

[12] Notomi T, Okayama H, Masubuchi H, Yonekawa T, Watanabe K, Amino N (2000). Loop-mediated isothermal amplification of DNA. Nucleic Acids Res.

[13] Tanner NA, Zhang Y, Evans TC (2015). Visual detection of isothermal nucleic acid amplification using pH-sensitive dyes. Biotechniques.

[14] FDA. Emergency use authorization (EUA) summary for the color SARS-CoV-2 RT-LAMP diagnostic assay. https://www.fda.gov/media/138249/download. 2021.

[15] Zhang Y, Odiwuor N, Xiong J, Sun L, Nyaruaba RO, Wei H (2020). Rapid molecular detection of SARS-CoV-2 (COVID-19) virus RNA using colorimetric LAMP. MedRxiv.

[16] Yu LWS, Hao X, Li X, Liu X, Ye S, Han H, Dong X, Li X, Li J, Liu N, Liu J, Zhang W, Pelechano V, Wei-Hua Chen W-H, Yin X (2020). Rapid colorimetric detection of COVID-19 coronavirus using a reverse tran- scriptional loop-mediated isothermal amplification (RT-LAMP) diagnostic plat- form: iLACO. MedRxiv.

[17] Hong TC, Mai QL, Cuong DV, Parida M, Minekawa H, Notomi T (2004). Development and evaluation of a novel loop-mediated isothermal amplification method for rapid detection of severe acute respiratory syndrome coronavirus. J Clin Microbiol.

[18] Coronavirus Disease 2019 (COVID-19). Centers for Disease Control and Prevention 2020.

[19] Mwananyanda L, Gill CJ, MacLeod W, Kwenda G, Pieciak R, Mupila Z (2021). Covid-19 deaths in Africa: prospective systematic postmortem surveillance study. BMJ..

[20] Mulenga LB, Hines JZ, Fwoloshi S, Chirwa L, Siwingwa M, Yingst S (2021). Prevalence of SARS-CoV-2 in six districts in Zambia in July, 2020: a cross-sectional cluster sample survey. Lancet Glob Health.

[21] Chaouch M (2021). Loop-mediated isothermal amplification (LAMP): An effective molecular point-of-care technique for the rapid diagnosis of coronavirus SARS-CoV-2. Rev Med Virol..

[22] Shabani E, Dowlatshahi S, Abdekhodaie MJ (2021). Laboratory detection methods for the human coronaviruses. Eur J Clin Microbiol Infect Dis.

[23] Baba MM, Bitew M, Fokam J, Lelo EA, Ahidjo A, Asmamaw K (2021). Diagnostic performance of a colorimetric RT-LAMP for the identification of SARS-CoV-2: a multicenter prospective clinical evaluation in sub-Saharan Africa. EClinicalMedicine.

[24] Schellenberg JJ, Ormond M, Keynan Y (2021). Extraction-free RT-LAMP to detect SARS-CoV-2 is less sensitive but highly specific compared to standard RT-PCR in 101 samples. J Clin Virol.

[25] Kaneko H, Kawana T, Fukushima E, Suzutani T (2007). Tolerance of loop-mediated isothermal amplification to a culture medium and biological substances. J Biochem Biophys Methods.

[26] Francois P, Tangomo M, Hibbs J, Bonetti EJ, Boehme CC, Notomi T (2011). Robustness of a loop-mediated isothermal amplification reaction for diagnostic applications. FEMS Immunol Med Microbiol.

[27] Chen HW, Ching WM (2016). The development of lyophilized loop-mediated isothermal amplification reagents for the detection of Coxiella burnetii. J Vis Exp..

[28] Kim D, Lee JY, Yang JS, Kim JW, Kim VN, Chang H (2020). The architecture of SARS-CoV-2 transcriptome. Cell.

[29] Promlek T, Thanunchai M, Phumisantiphong U, Hansirisathit T, Phuttanu C, Dongphooyao S (2022). Performance of colorimetric reverse transcription loop-mediated isothermal amplification as a diagnostic tool for SARS-CoV-2 infection during the fourth wave of COVID-19 in Thailand. Int J Infect Dis.

[30] Mautner L, Baillie CK, Herold HM, Volkwein W, Guertler P, Eberle U (2020). Rapid point-of-care detection of SARS-CoV-2 using reverse transcription loop-mediated isothermal amplification (RT-LAMP). Virol J.

[31] Woudenberg T, Eberle U, Marosevic D, Liebl B, Ackermann N, Katz K (2021). Detection and viral RNA shedding of SARS-CoV-2 in respiratory specimens relative to symptom onset among COVID-19 patients in Bavaria, Germany. Epidemiol Infect.

[32] Wolfel R, Corman VM, Guggemos W, Seilmaier M, Zange S, Muller MA (2020). Virological assessment of hospitalized patients with COVID-2019. Nature.

