# Supplementary material for: A fast extraction-free isothermal LAMP assay for detection of SARS-CoV-2 with potential use in resource-limited settings
Source: Virol J. 2022 May 2;19:77. doi: 10.1186/s12985-022-01800-7 (PMC9059459; doi:10.1186/s12985-022-01800-7)
Supplement: Supplementary file 1 — Additional file 1. [file 12985_2022_1800_MOESM1_ESM.docx]

**Supplementary Table S1:** Ct values and LAMP-results for samples from St. George’s Hospital; positive and negative results are depicted as 1 and 0, respectively.

| **Sample** | **Ct value N qRT-PCR** | **qRT-PCR N pos/neg** | **Ct value E qRT-PCR** | **qRT-PCR E pos/neg** | **Ct value S qRT-PCR** | **qRT-PCR S pos/neg** | **Orf-LAMP pos/neg** | **N-LAMP pos/neg** |
| --- | --- | --- | --- | --- | --- | --- | --- | --- |
| S01 | 16.52 | 1 | 17 | 1 | 17 | 1 | 1 | 1 |
| S02 | 16.63 | 1 | 17 | 1 | 16 | 1 | 1 | 1 |
| S03 | 17.65 | 1 | 18 | 1 | 18 | 1 | 1 | 1 |
| S04 | 18.74 | 1 | 19 | 1 | 19 | 1 | 1 | 1 |
| S05 | 19.63 | 1 | 20 | 1 | 19 | 1 | 1 | 1 |
| S06 | 20.23 | 1 | 21 | 1 | 20 | 1 | 1 | 1 |
| S07 | 20.27 | 1 | 20 | 1 | 19 | 1 | 1 | 1 |
| S08 | 20.58 | 1 | 21 | 1 | 20 | 1 | 1 | 1 |
| S09 | 20.76 | 1 | 21 | 1 | 20 | 1 | 1 | 1 |
| S10 | 20.88 | 1 | 22 | 1 | 21 | 1 | 1 | 1 |
| S11 | 21.31 | 1 | 22 | 1 | 21 | 1 | 1 | 1 |
| S12 | 21.63 | 1 | 22 | 1 | 22 | 1 | 1 | 1 |
| S13 | 22.50 | 1 | 23 | 1 | 22 | 1 | 1 | 1 |
| S14 | 22.50 | 1 | 23 | 1 | 22 | 1 | 1 | 1 |
| S15 | 23.32 | 1 | 23 | 1 | 22 | 1 | 1 | 1 |
| S16 | 23.81 | 1 | 24 | 1 | 23 | 1 | 1 | 1 |
| S17 | 24.22 | 1 | 24 | 1 | 24 | 1 | 1 | 1 |
| S18 | 25.33 | 1 | 26 | 1 | 25 | 1 | 1 | 1 |
| S19 | 26.14 | 1 | 26 | 1 | 25 | 1 | 1 | 1 |
| S20 | 26.39 | 1 | 26 | 1 | 26 | 1 | 1 | 1 |
| S21 | 26.49 | 1 | 26 | 1 | 25 | 1 | 1 | 1 |
| S22 | 26.52 | 1 | 27 | 1 | 26 | 1 | 1 | 1 |
| S23 | 26.85 | 1 | 27 | 1 | 26 | 1 | 1 | 1 |
| S24 | 26.99 | 1 | 27 | 1 | 25.91 | 1 | 1 | 1 |
| S25 | 27.52 | 1 | 27 | 1 | 27 | 1 | 1 | 1 |
| S26 | 28.06 | 1 | 27 | 1 | 27 | 1 | 1 | 1 |
| S27 | 28.95 | 1 | 29 | 1 | 28 | 1 | 0 | 1 |
| S28 | 29.33 | 1 | 29 | 1 | 29 | 1 | 1 | 1 |
| S29 | 29.36 | 1 | 30 | 1 | 29 | 1 | 1 | 1 |
| S30 | 29.80 | 1 | 30 | 1 | 30 | 1 | 0 | 1 |
| S31 | 30.41 | 1 | 29 | 1 | 29 | 1 | 0 | 1 |
| S32 | 30.74 | 1 | 29 | 1 | 29 | 1 | 0 | 0 |
| S33 | 31.29 | 1 | 31 | 1 | 30 | 1 | 0 | 0 |
| S34 | 31.31 | 1 | 31 | 1 | 30 | 1 | 0 | 0 |
| S35 | 31.84 | 1 | 30 | 1 | 29 | 1 | 0 | 1 |
| S36 | 32.20 | 1 | 28 | 1 | 27 | 1 | 0 | 1 |
| S37 | 32.29 | 1 | 32 | 1 | 32 | 1 | 0 | 0 |
| S38 | 32.89 | 1 | 30 | 1 | 29 | 1 | 0 | 0 |
| S39 | 34.28 | 1 | 32 | 1 | 31 | 1 | 0 | 0 |
| S40 | 34.70 | 1 | 32 | 1 | 31 | 1 | 0 | 0 |
| S41 | 34.89 | 1 | 31 | 1 | 30 | 1 | 0 | 0 |
| S42 | 35.16 | 1 | 31 | 1 | 30 | 1 | 0 | 0 |
| S43 | 35.40 | 1 | 32 | 1 | 31 | 1 | 0 | 0 |
| S44 | 35.85 | 1 | 33 | 1 | 33 | 1 | 0 | 0 |
| S45 | 35.88 | 1 | 32 | 1 | 31 | 1 | 0 | 0 |
| S46 | 39.26 | 0 | 33 | 1 | 32 | 1 | 0 | 0 |
| S47 | 39.51 | 0 | >40 | 0 | >40 | 0 | 0 | 0 |
| S48 | >40 | 0 | >40 | 0 | >40 | 0 | 0 | 0 |
| S49 | >40 | 0 | >40 | 0 | >40 | 0 | 0 | 0 |
| S50 | >40 | 0 | >40 | 0 | >40 | 0 | 0 | 0 |
| S51 | >40 | 0 | >40 | 0 | >40 | 0 | 0 | 0 |
| S52 | >40 | 0 | >40 | 0 | >40 | 0 | 0 | 0 |
| S53 | >40 | 0 | >40 | 0 | >40 | 0 | 0 | 0 |
| S54 | >40 | 0 | >40 | 0 | >40 | 0 | 0 | 0 |
| S55 | >40 | 0 | >40 | 0 | >40 | 0 | 0 | 0 |
| S56 | >40 | 0 | >40 | 0 | >40 | 0 | 0 | 0 |
| S57 | >40 | 0 | >40 | 0 | >40 | 0 | 0 | 0 |
| S58 | >40 | 0 | >40 | 0 | >40 | 0 | 0 | 0 |
| S59 | >40 | 0 | >40 | 0 | >40 | 0 | 0 | 0 |
| S60 | >40 | 0 | >40 | 0 | >40 | 0 | 0 | 0 |
| S61 | >40 | 0 | >40 | 0 | >40 | 0 | 0 | 0 |
| S62 | >40 | 0 | >40 | 0 | >40 | 0 | 0 | 0 |
| S63 | >40 | 0 | >40 | 0 | >40 | 0 | 0 | 0 |
| S64 | >40 | 0 | >40 | 0 | >40 | 0 | 0 | 0 |
| S65 | >40 | 0 | >40 | 0 | >40 | 0 | 0 | 0 |
| S66 | >40 | 0 | >40 | 0 | >40 | 0 | 0 | 0 |
| S67 | >40 | 0 | >40 | 0 | >40 | 0 | 0 | 0 |
| S68 | >40 | 0 | >40 | 0 | >40 | 0 | 0 | 0 |
| S69 | >40 | 0 | >40 | 0 | >40 | 0 | 0 | 0 |
| S70 | >40 | 0 | >40 | 0 | >40 | 0 | 0 | 0 |
| S71 | >40 | 0 | >40 | 0 | >40 | 0 | 0 | 0 |
| S72 | >40 | 0 | >40 | 0 | >40 | 0 | 0 | 0 |
| S73 | >40 | 0 | >40 | 0 | >40 | 0 | 0 | 0 |
| S74 | >40 | 0 | >40 | 0 | >40 | 0 | 0 | 0 |
| S75 | >40 | 0 | >40 | 0 | >40 | 0 | 0 | 0 |
| S76 | >40 | 0 | >40 | 0 | >40 | 0 | 0 | 0 |
| S77 | >40 | 0 | >40 | 0 | >40 | 0 | 0 | 0 |
| S78 | >40 | 0 | >40 | 0 | >40 | 0 | 0 | 0 |
| S79 | >40 | 0 | >40 | 0 | >40 | 0 | 0 | 0 |
| S80 | >40 | 0 | >40 | 0 | >40 | 0 | 0 | 0 |
| S81 | >40 | 0 | >40 | 0 | >40 | 0 | 0 | 0 |
| S82 | >40 | 0 | >40 | 0 | >40 | 0 | 0 | 0 |
| S83 | >40 | 0 | >40 | 0 | >40 | 0 | 0 | 0 |
| S84 | >40 | 0 | >40 | 0 | >40 | 0 | 0 | 0 |
| S85 | >40 | 0 | >40 | 0 | >40 | 0 | 0 | 0 |
| S86 | >40 | 0 | >40 | 0 | >40 | 0 | 0 | 0 |
| S87 | >40 | 0 | >40 | 0 | >40 | 0 | 0 | 0 |
| S88 | >40 | 0 | >40 | 0 | >40 | 0 | 0 | 0 |
| S89 | >40 | 0 | >40 | 0 | >40 | 0 | 0 | 0 |
| S90 | >40 | 0 | >40 | 0 | >40 | 0 | 0 | 0 |
| S91 | >40 | 0 | >40 | 0 | >40 | 0 | 0 | 0 |
| S92 | >40 | 0 | >40 | 0 | >40 | 0 | 0 | 0 |
| S93 | >40 | 0 | >40 | 0 | >40 | 0 | 0 | 0 |

**Supplementary Table S2:** Ct values and LAMP-results for samples from QECH; positive and negative results are depicted as 1 and 0, respectively.

| **Sample** | **Ct-value qRT-PCR** | **qRT-PCR pos/neg** | **N-LAMP pos/neg** |
| --- | --- | --- | --- |
| M01 | 14.43 | 1 | 1 |
| M02 | 16.24 | 1 | 1 |
| M03 | 18.17 | 1 | 1 |
| M04 | 20.86 | 1 | 1 |
| M05 | 21.08 | 1 | 1 |
| M06 | 21.63 | 1 | 1 |
| M07 | 22.06 | 1 | 1 |
| M08 | 22.11 | 1 | 1 |
| M09 | 22.34 | 1 | 1 |
| M10 | 23.16 | 1 | 1 |
| M11 | 23.80 | 1 | 1 |
| M12 | 25.37 | 1 | 1 |
| M13 | 25.75 | 1 | 1 |
| M14 | 26.49 | 1 | 1 |
| M15 | 26.89 | 1 | 1 |
| M16 | 27.42 | 1 | 1 |
| M17 | 27.46 | 1 | 1 |
| M18 | 28.67 | 1 | 0 |
| M19 | 29.16 | 1 | 1 |
| M20 | 29.55 | 1 | 1 |
| M21 | 31.28 | 1 | 1 |
| M22 | 31.61 | 1 | 1 |
| M23 | 31.82 | 1 | 1 |
| M24 | 32.10 | 1 | 1 |
| M25 | 32.16 | 1 | 0 |
| M26 | 32.34 | 1 | 1 |
| M27 | 32.38 | 1 | 1 |
| M28 | 33.00 | 1 | 1 |
| M29 | 33.64 | 1 | 0 |
| M30 | 38.50 | 1 | 0 |
| M31 | >40 | 0 | 1 |
| M32 | >40 | 0 | 0 |
| M33 | >40 | 0 | 0 |
| M34 | >40 | 0 | 0 |
| M35 | >40 | 0 | 0 |
| M36 | >40 | 0 | 0 |
| M37 | >40 | 0 | 0 |
| M38 | >40 | 0 | 0 |
| M39 | >40 | 0 | 0 |
| M40 | >40 | 0 | 0 |
| M41 | >40 | 0 | 0 |
| M42 | >40 | 0 | 0 |
| M43 | >40 | 0 | 0 |
| M44 | >40 | 0 | 0 |
| M45 | >40 | 0 | 0 |
| M46 | >40 | 0 | 0 |
| M47 | >40 | 0 | 0 |
| M48 | >40 | 0 | 0 |
| M49 | >40 | 0 | 0 |
| M50 | >40 | 0 | 0 |
| M51 | >40 | 0 | 0 |
| M52 | >40 | 0 | 0 |
| M53 | >40 | 0 | 0 |
| M54 | >40 | 0 | 0 |
| M55 | >40 | 0 | 0 |
| M56 | >40 | 0 | 0 |
| M57 | >40 | 0 | 0 |
| M58 | >40 | 0 | 0 |
| M59 | >40 | 0 | 0 |
| M60 | >40 | 0 | 0 |
| M61 | >40 | 0 | 0 |
| M62 | >40 | 0 | 0 |
| M63 | >40 | 0 | 0 |
| M64 | >40 | 0 | 0 |
| M65 | >40 | 0 | 0 |
| M66 | >40 | 0 | 0 |
| M67 | >40 | 0 | 0 |
| M68 | >40 | 0 | 0 |
| M69 | >40 | 0 | 0 |
| M70 | >40 | 0 | 0 |
| M71 | >40 | 0 | 0 |
| M72 | >40 | 0 | 0 |
| M73 | >40 | 0 | 0 |
| M74 | >40 | 0 | 0 |
| M75 | >40 | 0 | 0 |
| M76 | >40 | 0 | 0 |
| M77 | >40 | 0 | 0 |
| M78 | >40 | 0 | 0 |
| M79 | >40 | 0 | 0 |
| M80 | >40 | 0 | 0 |
| M81 | >40 | 0 | 0 |
| M82 | >40 | 0 | 0 |
| M83 | >40 | 0 | 0 |
| M84 | >40 | 0 | 0 |
| M85 | >40 | 0 | 0 |
| M86 | >40 | 0 | 0 |
| M87 | >40 | 0 | 0 |
| M88 | >40 | 0 | 0 |
| M89 | >40 | 0 | 0 |
| M90 | >40 | 0 | 0 |
| M91 | >40 | 0 | 0 |
| M92 | >40 | 0 | 0 |

**Supplementary Table S3:** Primers and probes for LAMP and qRT-PCR assays

| **Name** | **Target gene of primer set** | **Sequence** | **Reference** |
| --- | --- | --- | --- |
| ORF1a-A-F3 | *orf1a* | CTGCACCTCATGGTCATGTT | Zhang et al. (15) |
| ORF1a-A-B3 |  | AGCTCGTCGCCTAAGTCAA |  |
| ORF1a-A-FIP |  | GAGGGACAAGGACACCAAGTGTATGGTTGAGCTGGTAGCAGA |  |
| ORF1a-A-BIP |  | CCAGTGGCTTACCGCAAGGTTTTAGATCGGCGCCGTAAC |  |
| ORF1a-A-LF |  | CCGTACTGAATGCCTTCGAGT |  |
| ORF1a-A-LB |  | TTCGTAAGAACGGTAATAAAGGAGC |  |
| GeneN-A-F3 | *N* | TGGCTACTACCGAAGAGCT | Zhang et al. (15) |
| GeneN-A-B3 |  | TGCAGCATTGTTAGCAGGAT |  |
| GeneN-A-FIP |  | TCTGGCCCAGTTCCTAGGTAGTCCAGACGAATTCGTGGTGG |  |
| GeneN-A-BIP |  | AGACGGCATCATATGGGTTGCACGGGTGCCAATGTGATCT |  |
| GeneN-A-LF |  | GGACTGAGATCTTTCATTTTACCGT |  |
| GeneN-A-LB |  | ACTGAGGGAGCCTTGAATACA |  |
| Yu-F3 | *orf1ab* | CCACTAGAGGAGCTACTGTA | Yu et al. (16) |
| Yu-B3 |  | TGACAAGCTACAACACGT |  |
| Yu-FIP |  | AGGTGAGGGTTTTCTACATCACTATATTGGAACAAGCAAATTCTATGG |  |
| Yu-BIP |  | ATGGGTTGGGATTATCCTAAATGTGTGCGAGCAAGAACAAGTG |  |
| Yu-LF |  | CAGTTTTTAACATGTTGTGCCAACC |  |
| Yu-LB-3 |  | ATAGAGCCATGCCTAACATGCTTAG |  |
| SARS-F3 | *orf1b* | AACATGTTTATCACCCGCG | modified after Hong et al. (17) |
| SARS-B3 |  | CTCTGGAAAAATCTGTATT |  |
| SARS-FIP |  | GTAGCATGACACCCCTCGACAGAAGCTATAAGACATGT |  |
| SARS-BIP |  | GCTGTTGGTACCAATTTACCTATCAACATAACCTGTAGG |  |
| SARS-LF |  | CGAAGCCAATCCATGCA |  |
| SARS-LB |  | ACAGCTAGGTTTTTCTACAGG |  |
| nCoV19_CDC_N2-For | *N (qRT-PCR primers)* | TTACAAACATTGGCCGCAAA | CDC USA (5) |
| nCoV19_CDC_N2-Rev |  | GCGCGACATTCCGAAGAA |  |
| nCov19_CDC_N2_probe |  | 5'-HEX/ACAATTTGC/ZEN/CCCCAGCGCTTCAG/IABkFQ-3' |  |
