# Supplementary figures and images for: A fast extraction-free isothermal LAMP assay for detection of SARS-CoV-2 with potential use in resource-limited settings
Source: Virol J. 2022 May 2;19:77. doi: 10.1186/s12985-022-01800-7 (PMC9059459; doi:10.1186/s12985-022-01800-7)

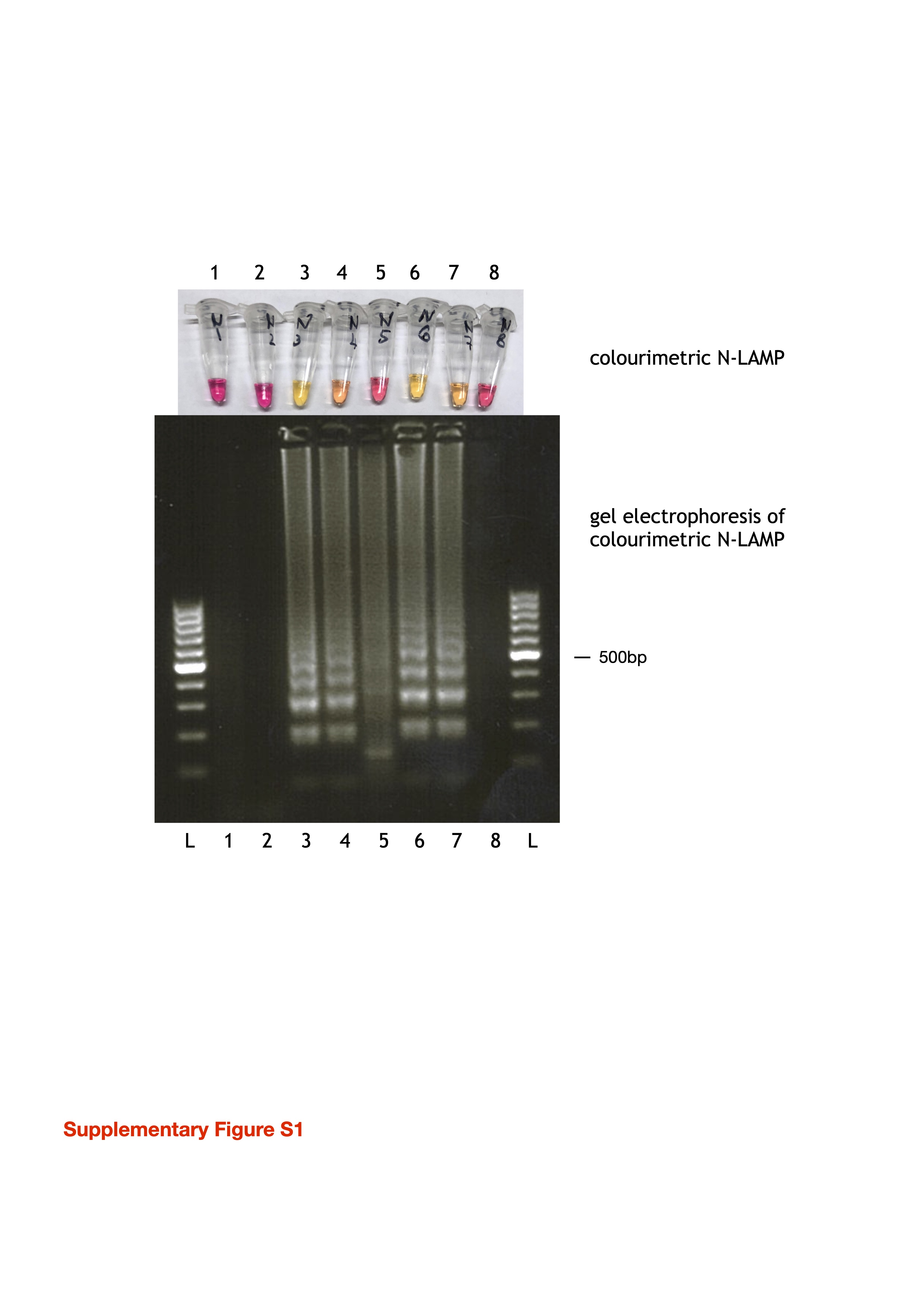

Supplement: Supplementary file 2 — Additional file 2: Figure S1. Colourimetric N-LAMP assay of positive and negative samples; and gel electrophoresis of colourimetric N-LAMP reaction. Lanes 3, 4, 6 and 7 show the typical ladder pattern of a positive LAMP reaction; 1—H2O control, 2—negative sample, extracted RNA, 3,4—positive samples, extracted RNA, 5—negative sample, extracted RNA, 6,7—positive samples, non-extracted RNA, 8—negative sample, non-extracted RNA, L—100 bp DNA ladder [file 12985_2022_1800_MOESM2_ESM.jpg]

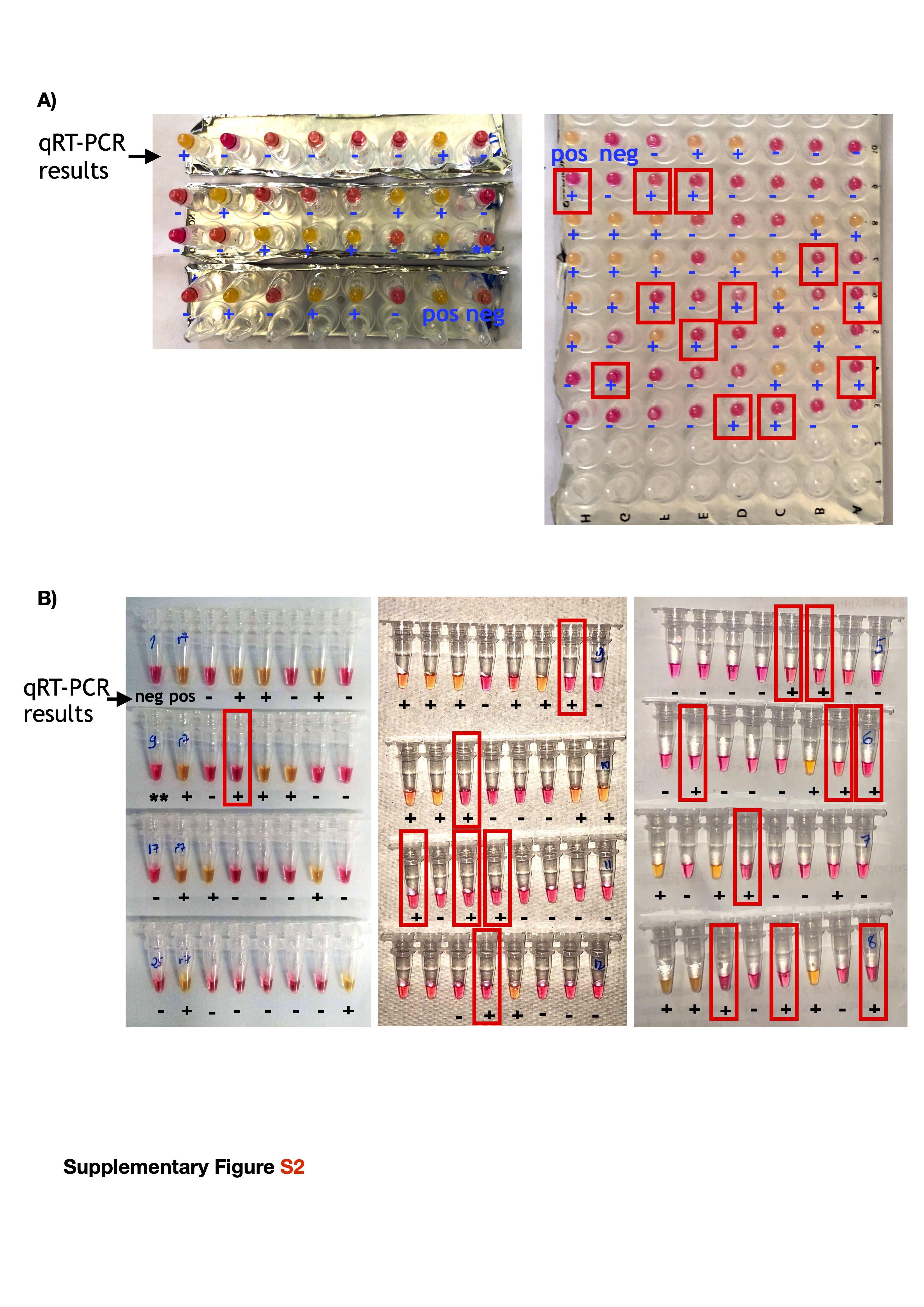

Supplement: Supplementary file 3 — Additional file 3: Figure S2. LAMP of 92 samples from St. George’s Hospital in comparison to qRT-PCR results (+/−); A N-LAMP (picture taken after 30 min, 63 °C) and B Orf-LAMP (picture taken after 40 min, 63 °C); discordant samples are depicted by a red square, − and + indicate negative and positive results in qRT-PCR; pos: positive control, neg: negative control, ** poor qRT-PCR curve/inconclusive [file 12985_2022_1800_MOESM3_ESM.jpg]
